# Supplementary figures and images for: A longitudinal analysis of associations between traffic-related air pollution with asthma, allergies and sensitization in the GINIplus and LISAplus birth cohorts
Source: PeerJ. 2013 Nov 7;1:e193. doi: 10.7717/peerj.193 (PMC3828611; doi:10.7717/peerj.193)

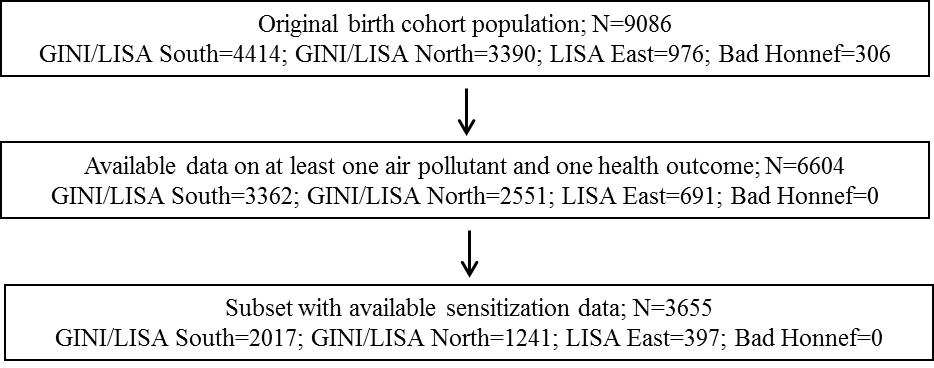

Supplement: Figure S1 [file peerj-01-193-s001.png]

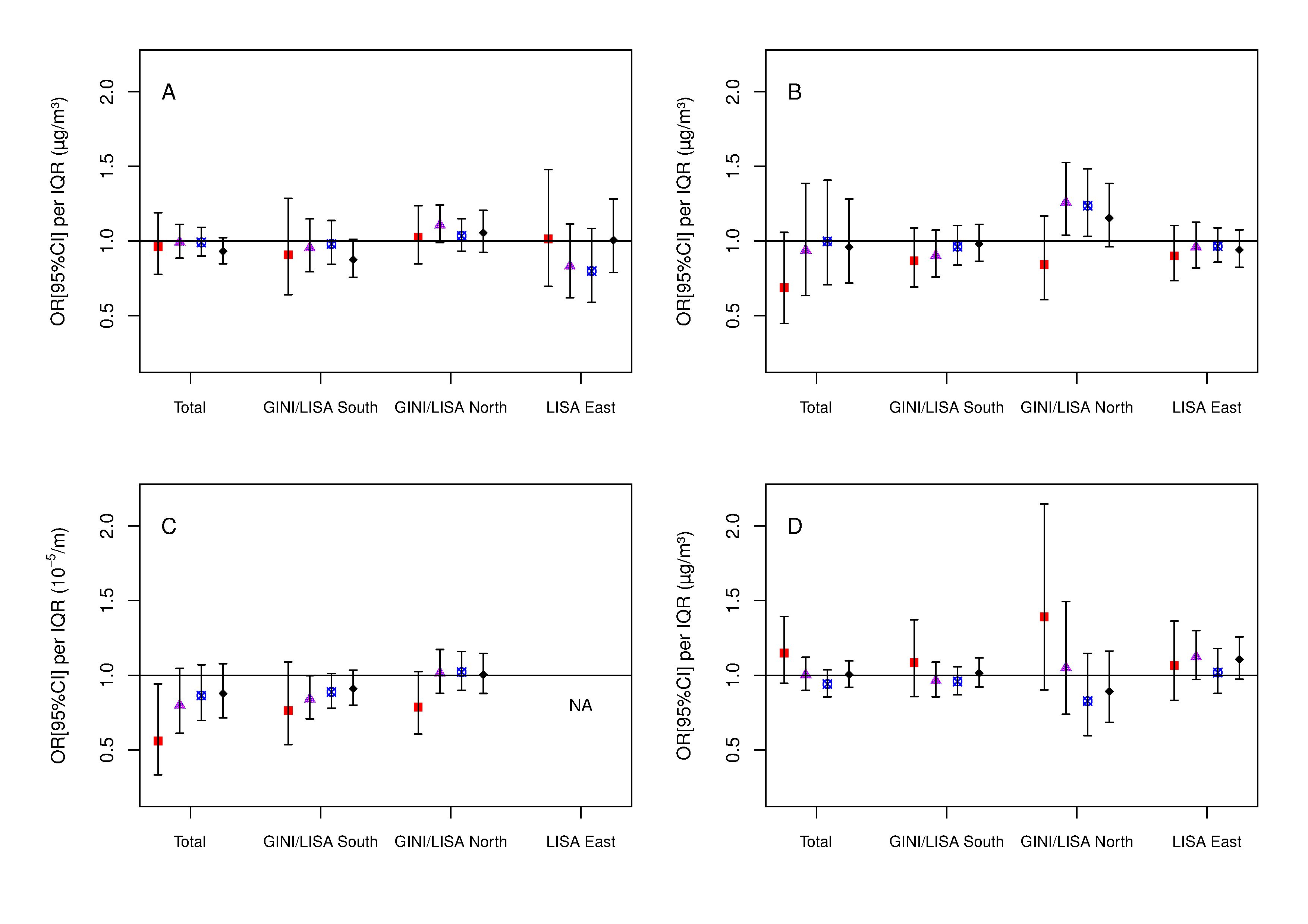

Supplement: Figure S2 — Total and area-specific associations between NO2 (A), PM2.5 mass (B), PM2.5 absorbance (C) and ozone (D) estimated at the age six-year address with doctor diagnosed asthma (red squares), doctor diagnosed allergic rhinitis (purple triangles), eye and nose symptoms (blue stars) and aeroallergen sensitization (black triangles). OR’s are calculated per interquartile increase of each air pollutant. Models are adjusted for sex, age, parental history of atopy, parental education, older siblings, maternal smoking during pregnancy, smoke exposure in the home, contact with furry pets, use of gas stove for cooking, home dampness or indoor mold, intervention participation, cohort and area (total models only). [file peerj-01-193-s002.png]

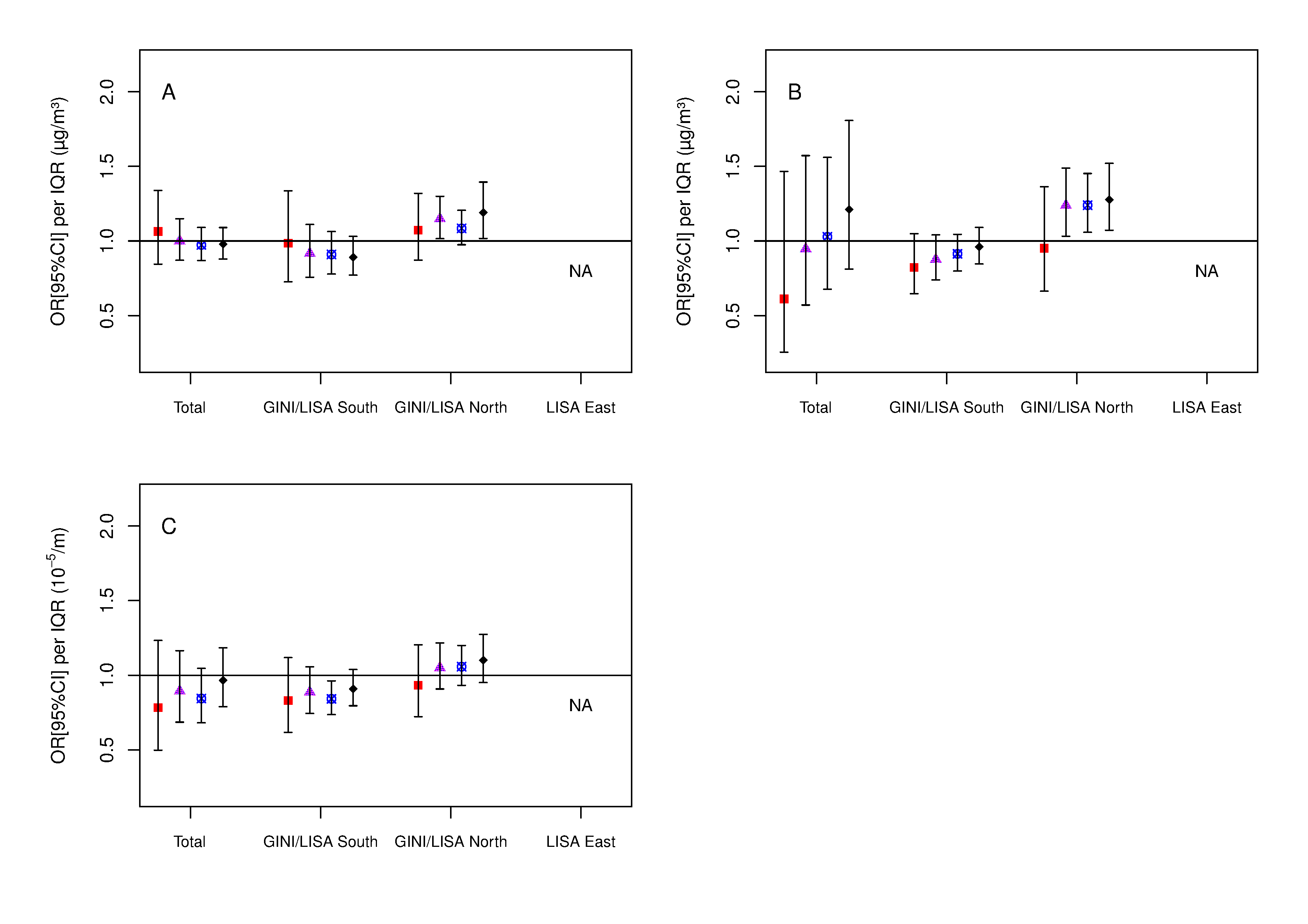

Supplement: Figure S3 — Total and area-specific associations between NO2 (A), PM2.5 mass (B) and PM2.5 absorbance (C) estimated at the age 10-year address with doctor diagnosed asthma (red squares), doctor diagnosed allergic rhinitis (purple triangles), eye and nose symptoms (blue stars) and aeroallergen sensitization (black triangles). OR’s are calculated per interquartile increase of each air pollutant. Models are adjusted for sex, age, parental history of atopy, parental education, older siblings, maternal smoking during pregnancy, smoke exposure in the home, contact with furry pets, use of gas stove for cooking, home dampness or indoor mold, intervention participation, cohort and area (total models only). [file peerj-01-193-s003.png]

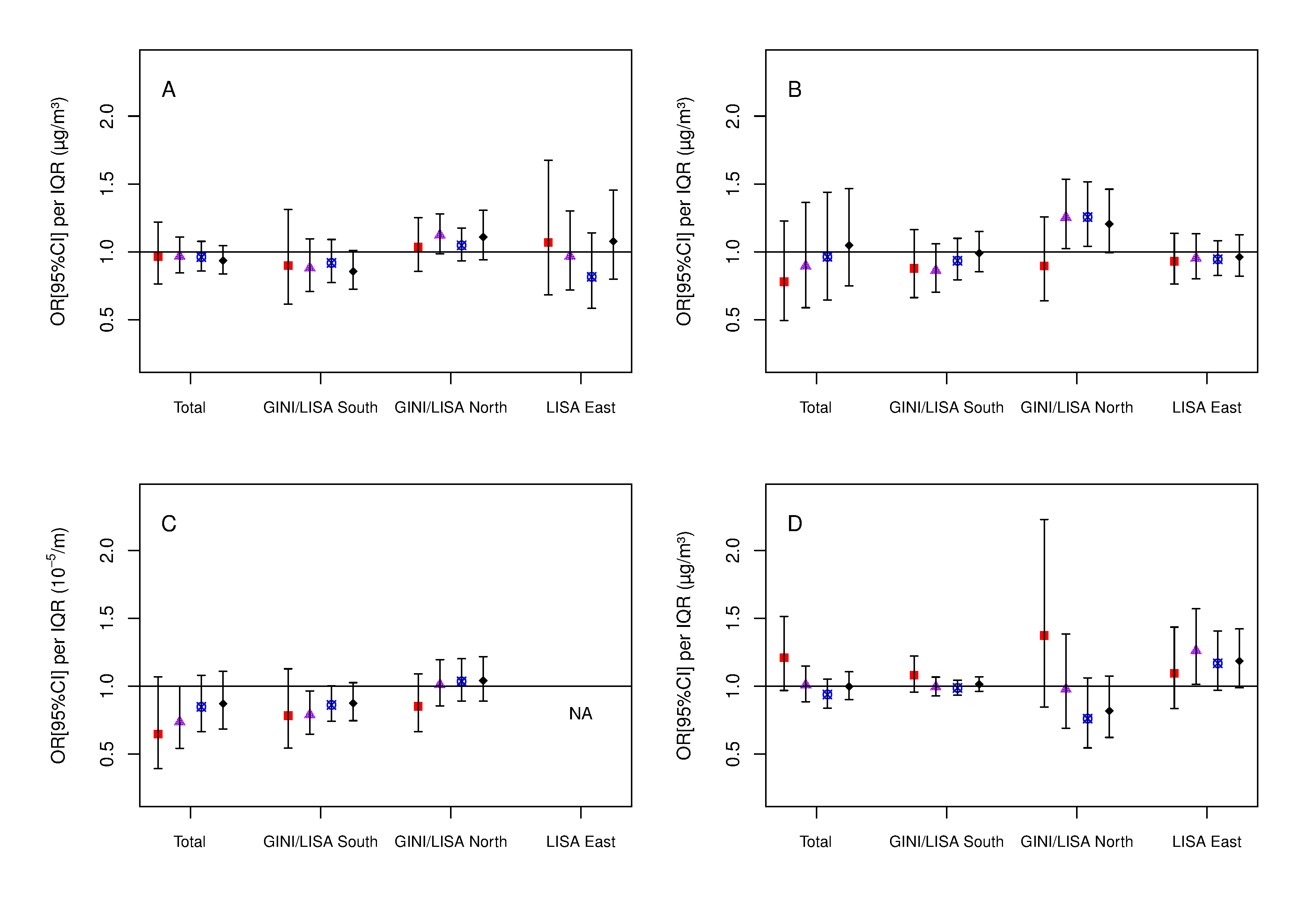

Supplement: Figure S4 — Total and area-specific associations between NO2 (A), PM2.5 mass (B) and PM2.5 absorbance (C) averaged at the birth, six and 10 year addresses, and ozone (D) averaged at the birth and six year addresses with doctor diagnosed asthma (red squares), doctor diagnosed allergic rhinitis (purple triangles), eye and nose symptoms (blue stars) and aeroallergen sensitization (black triangles). OR’s are calculated per interquartile increase of each air pollutant. Models are adjusted for sex, age, parental history of atopy, parental education, older siblings, maternal smoking during pregnancy, smoke exposure in the home, contact with furry pets, use of gas stove for cooking, home dampness or indoor mold, intervention participation, cohort and area (total models only). [file peerj-01-193-s004.png]
