# Supplementary material for: A longitudinal analysis of associations between traffic-related air pollution with asthma, allergies and sensitization in the GINIplus and LISAplus birth cohorts
Source: PeerJ. 2013 Nov 7;1:e193. doi: 10.7717/peerj.193 (PMC3828611; doi:10.7717/peerj.193)
Supplement: Table S1 — Characteristics of study participants with available serology data (N = 3655). [file peerj-01-193-s005.docx]

**Supplemental Table S1:** Characteristics of study participants with available serology data (N=3655)

| **General characteristics** | | **n/N** | **%** |
| --- | --- | --- | --- |
| Males | | 1891/3655 | 51.7 |
| Presence of older siblings | | 1679/3648 | 46.0 |
| Parental education Less than or equal to 10 years | | 1204/3640 | 33.1 |
|  | More than 10 years | 2436/3640 | 66.9 |
| Smoking During pregnancy | | 487/3581 | 13.6 |
|  | Ever in home (1-4 years) | 1314/3473 | 37.8 |
| Parental history of atopy | | 2229/3621 | 61.6 |
| Owned furry pet during early life | | 590/3532 | 16.7 |
| Gas used in home during early life | | 270/3597 | 7.5 |
| Mold/dampness in home during early life | | 855/3341 | 25.6 |
| Moved between one and 10 years | | 1882/3296 | 57.1 |
| Area | GINI/LISA South | 2017/3655 | 55.2 |
|  | GINI/LISA North | 1241/3655 | 34.0 |
|  | LISA East | 397/3655 | 10.9 |
| Cohort GINIplus | | 2388/3655 | 65.3 |
|  | LISAplus | 1267/3655 | 34.7 |
| Intervention participation^1^ | | 1223/3655 | 33.5 |
| **Health outcomes (at age 10 years)** | |  |  |
| Doctor diagnosed asthma | | 137/3208 | 4.3 |
| Doctor diagnosed allergic rhinitis | | 357/3168 | 11.3 |
| Eyes and nose symptoms | | 497/3242 | 15.3 |
| Sensitized to aeroallergens | | 1100/2735 | 40.2 |
| Sensitized to indoor aeroallergens | | 748/2732 | 27.4 |
| Sensitized to outdoor aeroallergens | | 809/2734 | 29.6 |

^1^Intervention only part of the GINIplus cohort
